# Nuclear Factor Erythroid 2-related Factor 2 Deficiency Exacerbates Lupus Nephritis in B6/*lpr* mice by Regulating Th17 Cell Function

**DOI:** 10.1038/srep38619

**Published:** 2016-12-12

**Authors:** Mei Zhao, Huanpeng Chen, Qingfeng Ding, Xiaoxie Xu, Bolan Yu, Zhaofeng Huang

**Affiliations:** 1 Institute of Human Virology, Zhongshan School of Medicine, Sun Yat-sen University, Guangzhou, China; 2 Key Laboratory of Tropical Diseases Control, Sun Yat-sen University, Ministry of Education in China, Guangzhou, China; 3 Key Laboratory for Major Obstetric Diseases of Guangdong Province, Third Affiliated Hospital of Guangzhou Medical University, Guangzhou, China; 4 Department of Biochemistry, Zhongshan School of Medicine, Sun Yat-sen University, Guangzhou, China.

## Abstract

Lupus nephritis (LN) is the major clinical manifestation of systemic lupus erythematosus. LN is promoted by T helper 17 (Th17) cells, which are the major pro-inflammatory T cell subset contributing to autoimmunity regulation. Nuclear factor erythroid 2-related factor 2 (NRF2) is critical for suppressing reactive oxygen species (ROS) and relieving oxidant stress by regulating antioxidant gene expression. Previous studies have demonstrated that *Nrf2* deficiency promotes drug-induced or spontaneous LN. However, whether NRF2 regulates Th17 function during LN development is still unclear. In this study, we introduced *Nrf2* deficiency into a well-known LN model, the B6/*lpr* mouse strain, and found that it promoted early-stage LN with altered Th17 activation. Th17 cells and their relevant cytokines were dramatically increased in these double-mutant mice. We also demonstrated that naïve T cells from the double-mutant mice showed significantly increased differentiation into Th17 cells *in vitro*, with decreased expression of the Th17 differentiation suppressor *Socs3* and increased phosphorylation of STAT3. Our results demonstrated that *Nrf2* deficiency promoted Th17 differentiation and function during LN development. Moreover, our results suggested that the regulation of Th17 differentiation via NRF2 could be a therapeutic target for the treatment of subclinical LN patients.

Lupus nephritis (LN) is the major clinical manifestation of systemic lupus erythematosus (SLE). SLE is a complicated autoimmune disease that is characterised by the production of autoantibodies, systemic inflammation, and damage to vessels and organs^1^. SLE is a multifactorial disease caused by genetic and environmental factors. Many SLE susceptibility genes are responsible for maintaining immune tolerance and homeostasis, such as antigen processing and presentation, clearance of apoptotic debris, leukocyte cell surface receptors, and cell signalling and gene transcription molecules^2^,^3^,^4^. The pathogenesis of LN involves abnormal B and T cell responses, which promote the production of autoantibodies and immune complex deposits in the kidney and other organs^5^. Recent studies have found that T cells are primarily responsible for the pathogenesis of LN, including the regulation of B cell responses and the production of autoantibodies, the modulation and differentiation of T helper (Th) cell and effector cell expansion and function, and the activation of macrophages and natural killer cell functions^5^,^6^.

Th cells, which are central regulators of adaptive immune responses, play a crucial role in the pathogenesis of SLE by regulating the interactions between other cells and contributing to the production of immunomodulatory cytokines^4^. Upon antigen stimulation, naïve CD4^+^ T cells differentiate into different lineages of Th cells, including Th1, Th2, Th17, Th9, Th22, and Treg cells, according to the types of cytokines they are stimulated by^7^. In particular, Th17 cells, which produce the pro-inflammatory cytokine interleukin (IL)-17, are important for the pathogenesis of SLE^8^,^9^,^10^. Patients with SLE have higher levels of IL-17 in serum compared to healthy controls, and an increased amount of IL-17-producing T cells in peripheral blood^11^,^12^,^13^. B6/*lpr* mice deficient in IL-23 signalling were resistant to the development of LN and showed deficient Th17 development^14^. Th17 also co-regulates the pathogenesis of SLE together with type I interferon^15^, which regulates the germinal centre reactions by IL-21- and IL-17-dependent follicular Th cells in BXD2 mice^16^,^17^,^18^. It is well known that Th17 cells regulate SLE pathogenesis through multiple mechanisms^19^.

Nuclear factor erythroid 2-related factor 2 (NRF2) is a basic leucine zipper transcription factor and is essential for protecting cells against oxidative stress^20^,^21^. NRF2 acts through the antioxidant response element (ARE)/electrophile response element (EpRE) to regulate the expression of antioxidative enzymes and coordinate a wide range of responses to oxidative damage resulting from electrophiles and reactive oxygen species (ROS)^20^,^21^. *Nrf2* gene variation is associated with LN in childhood-onset SLE^22^. A GWAS analysis also defined the *Nrf2* locus as a region associated with susceptibility to SLE^23^. *Nrf2*-null mice developed a lupus-like nephritis syndrome in an age-dependent manner or when treated with pristane induction methods^24^,^25^,^26^. Prior studies from both human and murine models reported that NRF2 was involved in the pathogenesis of SLE, but the function of NRF2 in the development of LN is still unclear.

To further understand the mechanisms of how immune homeostasis and immune compounds are affected by *Nrf2* deficiency, and how *Nrf2* deficiency increases susceptibility to LN, we produced *Nrf2*^−/−^*lpr*/*lpr* mice with a C57BL/6 background (designated as B6.*Nrf2*^−/−^*lpr*/*lpr*) to observe the clinical development of LN. The B6/*lpr* mice, which carried a *Fas* gene mutation and a defect in FAS-mediated apoptosis, developed lupus-like autoimmune manifestations^27^. We found that the B6.*Nrf2*^−/−^*lpr*/*lpr* mice developed significant LN with an increased abundance of Th17 cells and a raised level of serum IL-17. Our results suggested that elevated oxidative damage exacerbated the development of LN by promoting the differentiation of Th17 cells and augmenting the production of cytokines such as IL-17, IL-23, and IL-1β in B6.*Nrf2*^−/−^*lpr*/*lpr* mice.

## Results

### Survival rates

**Figure 1:**
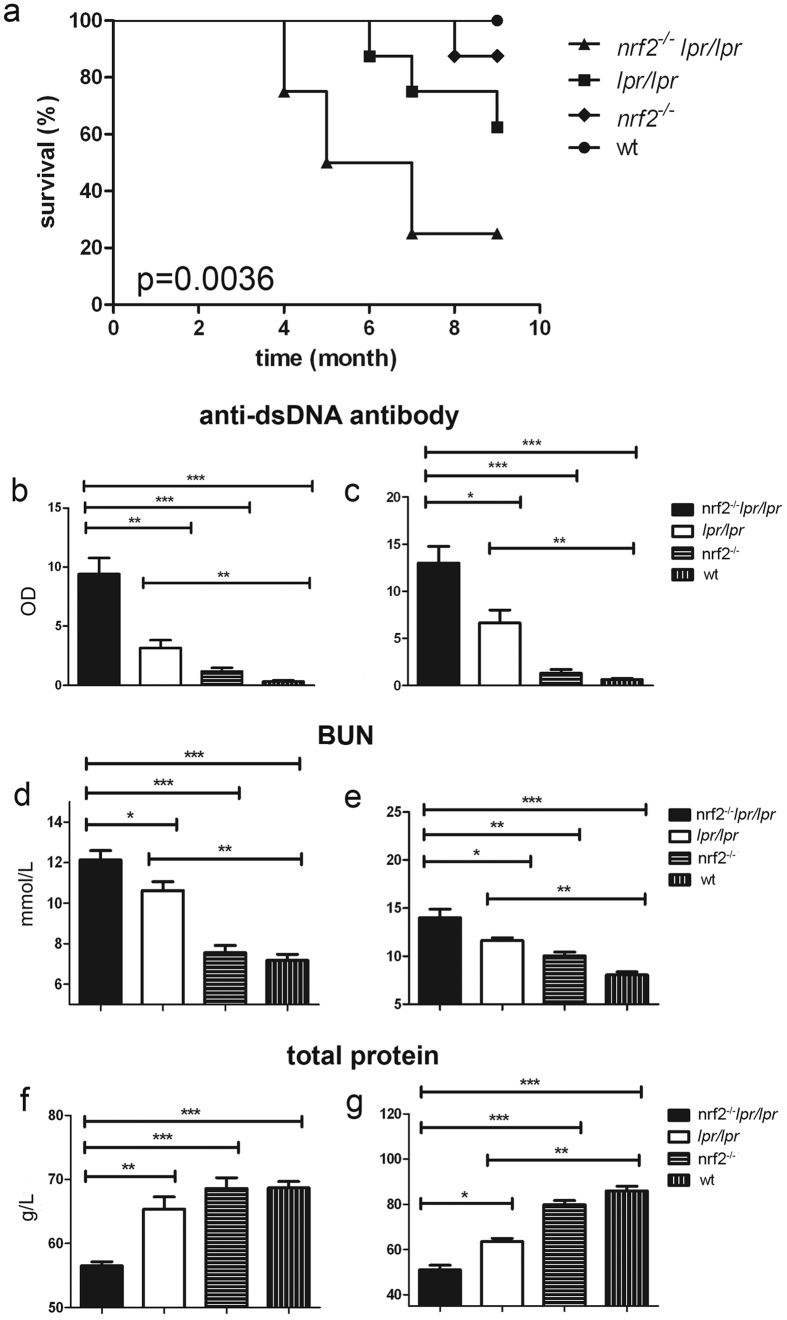
Survival rates, renal function, and anti-dsDNA autoantibody production. During the course of 9 months, the survival of mice with different genotypes was monitored (**a**). Enzyme-linked immunosorbent assay analyses of the serum anti-dsDNA antibody level (*n* = 11) were carried at 3 months (**b**) and 6 months (**c**) of age. To analyse the development of renal insufficiency, we measured the serum levels of blood urea nitrogen (**d** and **e**) and total protein (**f** and **g**) at different times (*n* = 8). Data values represent mean ± SEM. *p < 0.05, **p < 0.01, ***p < 0.001.

Female B6.*Nrf2*^−/−^*lpr*/*lpr* mice had markedly shorter lifespans than wild-type (WT) mice. At 6 months of age they showed 50% (4/8) survival, while 87.5% (7/8) of the B6.*lpr*/*lpr* mice and all of the B6.*Nrf2*^−/−^ and WT mice survived at that age. By the age of 9 months, 87.5% (7/8) of the B6.*Nrf2*^−/−^*lpr/lpr* mice had died, compared with 37.5% (3/8) of the B6.*lpr/lpr* mice, 12.5% (1/8) of the B6.*Nrf2*^−/−^ mice, and none of the WT mice (Fig. 1a). The B6.*Nrf2*^−/−^*lpr/lpr* mice died at younger ages than the other three genotypes of mice with statistical significance (*p* = 0.0036).

### Renal function and anti-dsDNA autoantibody production

The production of autoantibodies and their deposition in various locations are the most common indicators of LN. In female B6.*Nrf2*^−/−^*lpr*/*lpr* mice, the production of anti-dsDNA antibodies was significantly higher than in the other three genotypes, and was first observed once the mice were 3 months old (Fig. 1b). At 6 months of age, the B6.*Nrf2*^−/−^*lpr*/*lpr* mice had higher levels of antibodies than they did at 3 months of age, which was consistent with prior studies showing that anti-dsDNA antibody production in LN increased with age (Fig. 1c). We also found that anti-dsDNA antibody production in the B6/*lpr* mice increased, but the B6.*Nrf2*^−/−^ mice showed no differences from the WT mice at 6 months of age, which was in accordance with prior studies^24^.

In our study, the level of serum blood urea nitrogen (BUN) increased significantly in the B6.*Nrf2*^−/−^*lpr*/*lpr* mice compared with the mice of the other three genotypes at 3 months (Fig. 1d) and 6 months of age (Fig. 1e), and displayed typical azotemia with BUN values of 12.13 ± 0.4605 mmol/L at 3 months and 14.00 ± 0.8964 mmol/L at 6 months. Other mice had nearly normal BUN values, except for the B6/*lpr* mice aged 6 months, which reached the clinical standard of azotemia (11.63 ± 0.295 mmol/L). These results were different from ICR background mice with the same genotypes^28^, indicating that the effects of *Nrf2* deficiency on disease pathogenesis and susceptibility vary with the genetic background. The level of total protein in serum was also significantly lower in the B6.*Nrf2*^−/−^*lpr*/*lpr* mice than in the other three genotypes at 3 months (Fig. 1f) and 6 months (Fig. 1g), indicating that protein was lost from the body as a result of renal function dysregulation in the mutant mice.

### Histological analysis and immune complex deposition in kidney

**Figure 2:**
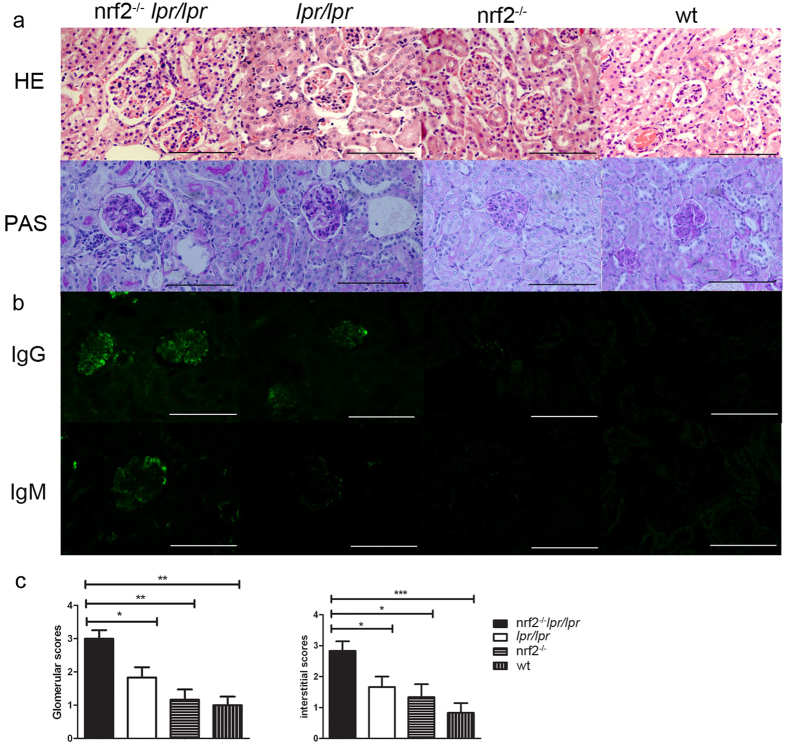
Histopathologic analysis of renal tissues. Representative photomicrographs of kidneys from B6.*Nrf2*^−/−^*lpr*/*lpr*, B6/*lpr*, B6.*Nrf2*^−/−^, and WT B6 mice (*n* = 6) at 6 months old are shown. The sections were stained with H&E and PAS (**a**) All images were taken at 400× total magnification. Glomerular injury was scored from 0 to 4 and interstitial injury from 0 to 4. Shown are scores for the mice of each genotype at 6 months old (**c**). (**b**) Immunostaining of immunocomplex deposition (IgG and IgM) in kidney at 6 months old. Data values represent mean ± SEM. *p < 0.05, **p < 0.01, ***p < 0.001.

Histomorphologic changes in kidneys with HE and PAS staining were performed in 6-month-old female mice. The B6.*Nrf2*^−/−^*lpr/lpr* mice had a remarkable change in glomerular histology, including increased mesangial expansion, basement membrane thickening, and increased interstitial infiltration with the formation of sclerotic crescents and lymphocytic infiltration (Fig. 2a). We evaluated the severity of renal impairment using the following score criterion: (1) four points for severe diffuse proliferation, with sclerotic crescent formation in glomeruli and multifocal infiltrates with extensive necrosis in interstitial tissue; (2) two to three points for diffuse and focal proliferation in glomeruli and multifocal infiltrates in interstitial tissue; and (3) one point for focal, mild, or early proliferation in glomeruli and occasional infiltrates in interstitial tissue. The statistical analysis of renal impairment scoring showed that the deficiency of *Nrf2* in the B6*/lpr* mice significantly aggravated the severity of histological changes associated with LN in both the glomerular and interstitial tissues (Fig. 2c).

The detection of immune complex deposition in the kidney was more observable when the IgG and IgM deposition complexes accumulated in glomeruli, and there was more thickening of the glomerular membrane in the B6.*Nrf2*^−/−^*lpr/lpr* mice than in the B6*/lpr* mice. Almost no IgG and IgM complexes were detected in the B6.*Nrf2*^−/−^ and WT mice (Fig. 2b). This result is consistent with the serum levels of anti-dsDNA antibodies and the results of histological analysis.

### Total number of lymphocytes and proportions of lymphocyte subsets in spleen

**Figure 3:**
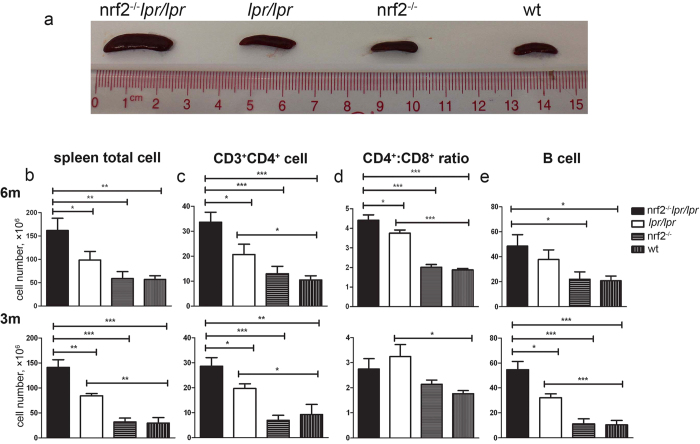
Composition of lymphocytes in spleen. Spleens and splenic lymphocytes were isolated from B6.*Nrf2*^−/−^*lpr*/*lpr*, B6/*lpr*, B6.*Nrf2*^−/−^, and WT B6 mice at 6 months old (*n* = 9) and 3 months old (*n* = 7) and counted, then analysed by flow cytometry. (**a**) The spleens of the *Nrf2*^−/−^*lpr*/*lpr* mice were the largest in comparison with those of other genotypes of mice at 6 months old. (**b**) The total number of lymphocytes in the B6.*Nrf2*^−/−^*lpr*/*lpr* mice were increased at both 3 and 6 months. The proportion of CD3^+^CD4^+^ T cells was significantly increased in the B6.*Nrf2*^−/−^*lpr*/*lpr* mice compared with that in the B6.*lpr* mice (**c**). (**d**) At 6 months of age, in the B6.*Nrf2*^−/−^*lpr*/*lpr* mice, the proportions of CD3^+^CD4^+^ T cells and CD3^+^CD8^+^ T cells among splenic lymphocytes were higher than those in the other mouse genotypes. The *Nrf2*^−/−^*lpr*/*lpr* mice had more B cells in spleen (**e**). Data values represent mean ± SEM. *p < 0.05, **p < 0.01, ***p < 0.001.

As previously reported^29^, one of the clinical characteristics of the B6*/lpr* mice is splenomegaly. We conducted a post-mortem analysis and found that the spleens of the B6*/lpr* mice were larger than those of the *Nrf2*^−/−^ and WT mice at 6 months of age, and that *Nrf2* deficiency dramatically aggravated the splenomegaly in B6/*lpr* mice (Fig. 3a). The total number of lymphocytes in the spleen was also significantly increased in the B6.*Nrf2*^−/−^*lpr*/*lpr* mice (Table 1 and Fig. 3b).

We investigated the composition of splenic lymphocytes by flow cytometry, which demonstrated that the proportion of CD3^+^CD4^+^ T cells among splenic CD4^+^ T cells was markedly elevated in the B6.*Nrf2*^−/−^*lpr*/*lpr* mice (Fig. 3c and Table 1). However, no significant differences were observed in the proportions of CD3^+^CD8^+^ T cells among different genotypes (Table 1). The ratio of the CD4^+^ and CD8^+^ T cells among total splenic lymphocytes increased significantly because of the increased cell count in the B6.*Nrf2*^−/−^*lpr*/*lpr* mice (Fig. 3d). The B cell number also increased in the B6.*Nrf2*^−/−^*lpr*/*lpr* mice compared to the other three genotypes (Fig. 3e). These results demonstrated that an *Nrf2* null mutation increased the abundance of several different lymphocyte subsets in the B6/*lpr mice*, which demonstrated the role of these cells in exacerbating LN in the B6.*Nrf2*^−/−^*lpr*/*lpr* mice.

### Cytokine secretion in the peripheral blood

**Figure 4:**
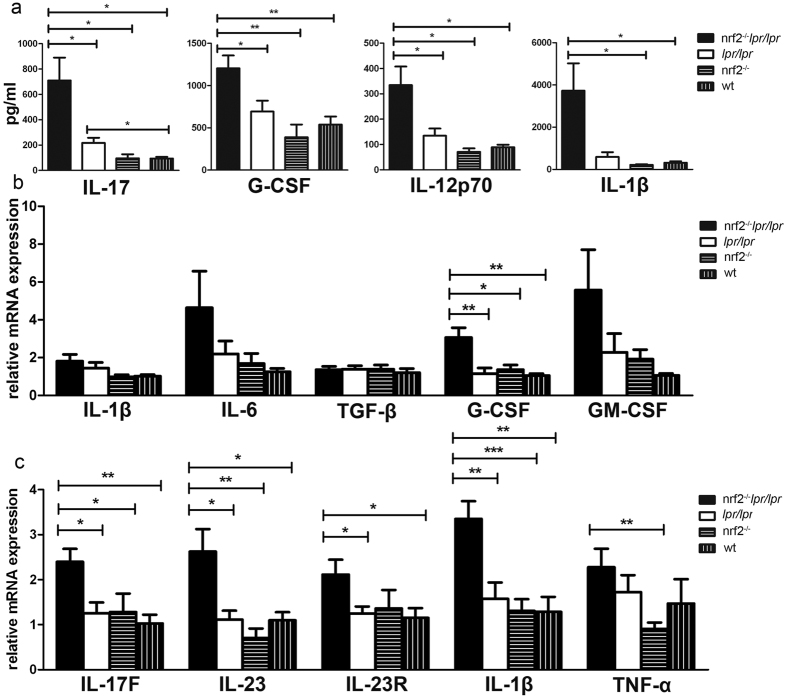
Cytokine levels in the peripheral blood, kidney, and macrophages. (**a**) The levels of IL-17, G-CSF, IL-12p7, and IL-1β in serum as determined by a Bio-Plex Pro™ Mouse Cytokine 23-Plex Panel. (**b**) Cytokine expression in macrophages activated using lipopolysaccharides as measured by qRT-PCR. (**c**) The expression of Th17-related cytokines in kidneys as determined by qRT-PCR. Mice were aged 6 months. *n* = 6 for each group. Data values represent mean ± SEM. *p < 0.05, **p < 0.01, ***p < 0.001.

We detected 23 cytokines in serum using the multiplex method, including IL-1β, IL-12p70, G-CSF, and IL-17. Significant changes were displayed among the different genotype mice at 6 months of age (Fig. 4a). Among these cytokines, the serum levels of IL-17, G-CSF, and IL-12p70 increased significantly in the B6.*Nrf2*^−/−^*lpr*/*lpr* mice compared to the other mice (Fig. 4a). IL-1β was detected at a higher concentration in the B6.*Nrf2*^−/−^*lpr*/*lpr* mice than in the other mice. However, significant differences were only detected between the B6.*Nrf2*^−/−^*lpr*/*lpr* mice and WT or *Nrf2*-null mice, while there was no significant difference between the B6.*Nrf2*^−/−^*lpr*/*lpr* and B6/*lpr* mice. This lack of significance may have been due to the high variation among individual mice (Fig. 4a). Other cytokines including IL-1α, IL-2, IL-3, IL-4, IL-5, IL-6, IL-10, IL-13, GM-CSF, IFN-γ, KC, MCP-1, MIP-1α, MIP-1β, RANTES, TNF-α, and IL-9 displayed no significant differences among the B6.*Nrf2*^−/−^*lpr*/*lpr* mice and the other three genotypes.

### Cytokine expression of macrophages

Previous studies demonstrated that *Nrf2*-deficient macrophages had an impaired antigen-presenting function and an impaired antigen-induced T cell function with altered cytokine production^30^. To evaluate whether similar changes occurred in the mouse genotypes investigated in this study, the mRNA levels of a number of inflammatory factors in macrophages were measured, and the results showed that *Csf3* expression increased significantly in the B6.Nrf2^−/−^*lpr*/*lpr* mice (Fig. 4b). Several cytokines including *Il1b, Il6*, and *Csf2* demonstrated slight increases in expression, but these differences did not reach statistical significance in the B6.*Nrf2*^−/−^*lpr*/*lpr* mice (Fig. 4b).

### Th17 cells and Th17-associated cytokines production in spleen and kidney

**Figure 5:**
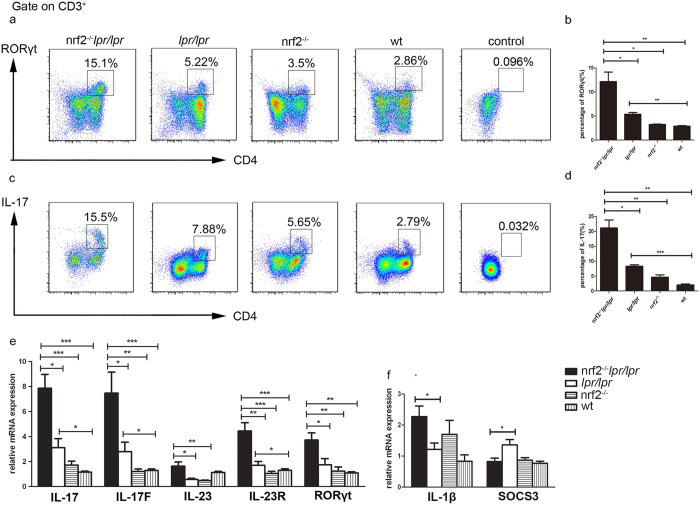
Splenic Th17 cell population and cytokine expression. Lymphocytes were collected from B6.*Nrf2*^−/−^*lpr*/*lpr (n* = 4), B6/*lpr (n* = 4), B6.*Nrf2*^−/−^ (*n* = 3), and WT B6 (*n* = 4) mice at 6 months of age and activated with phorbol 12-myristate 13-acetate (50 ng/mL) and ionomycin (1 μg/mL). (**a** and **b**) Representative flow cytometry plots and data showing a higher proportion of RORγt^+^CD3^+^CD4^+^Th17 cells among splenic CD4^+^ cells in the *Nrf2*^−/−^*lpr*/*lpr* mice than in the mice of other genotypes. (**c**) Representative flow cytometry plots demonstrating interleukin-17A (IL-17A) production by CD3^+^CD4^+^ T cells from mice of four different genotypes. (**d**) The percentage of T cells producing IL-17A was markedly increased in the *Nrf2*^−/−^*lpr*/*lpr* mice. (**e** and **f**) The mRNA levels of cytokines associated with Th17 in splenic cells. Data values represent mean ± SEM. *p < 0.05, **p < 0.01, ***p < 0.001.

Our analysis of cytokines in serum revealed that the concentration of IL-17 in serum changed in the B6.*Nrf2*^−/−^*lpr*/*lpr* mice, which indicated a change in the function of the Th17 cells. To confirm this observation, we examined the proportion of Th17 cells among splenic CD4^+^ T cells by intracellular staining and flow cytometry, and found that the proportion of RORγt^+^CD3^+^CD4^+^ T cells dramatically increased in the B6.*Nrf2*^−/−^*lpr*/*lpr* mice (Fig. 5a and b), as did that of IL-17-producing CD3^+^CD4^+^ T cells (Fig. 5c and d).

The mRNA levels of characteristic Th17 cytokines and molecules including *Il17, Il17f, Il23, Il23r*, and *Rorγt* increased significantly in the B6.*Nrf2*^−/−^*lpr*/*lpr* mice as determined by a quantitative real time polymerase chain reaction (qRT-PCR) analysis (Fig. 5e). In addition, the mRNA levels of the inflammatory cytokine genes *Il1b* and *Socs3* in the spleen were significantly changed in the B6.*Nrf2*^−/−^*lpr*/*lpr* mice (Fig. 5f), which would be expected to be important for the activity of STAT3 and the early differentiation of Th17 cells. These results indicated increased Th17 cell immune responses during the aggravation of LN in the B6.*Nrf2*^−/−^*lpr*/*lpr* mice.

It is unclear whether Th1 cells participate in the exacerbation of LN since only the transcripts of IL-12B p70 slightly increased in the B6.*Nrf2*^−/−^*lpr*/*lpr* mice relative to the mice of other genotypes. To confirm our results, we also measured the mRNA expression of cytokines and key transcription factors related to Th1 cells and other Th cell subsets, including *Il2, Ifng, Il4, Il10, Il13, Tgfb, Bcl6, Prdm1, Foxp3, Tbx21, Gata3, Il6, Il9, Hif1, Il21*, and *Csf2*, and observed no significant differences between the B6.*Nrf2*^−/−^*lpr*/*lpr* mice and the other mice.

We also measured the mRNA expression of Th17-related cytokines in kidney to investigate the role of lymphocytes in the lupus-associated kidney damage. The expression of most of the Th17-associated cytokines besides *Il17*, including *Il17f, Il23, Il23r*, and *Il1b*, increased significantly in the B6.*Nrf2*^−/−^*lpr*/*lpr* mice (Fig. 4c). *Il17* could not be detected in the renal RNA sample because of the limitations of the qRT-PCR detection limit. These results indicated that more Th17 cells infiltrated into the kidneys of the B6.*Nrf2*^−/−^*lpr*/*lpr* mice than in the other mice, which led to more serious damage in the kidney with increased inflammation.

### *In vitro* Th17 cell differentiation increased in B6.*Nrf2* ^−/−^ *lpr*/*lpr* mice

**Figure 6:**
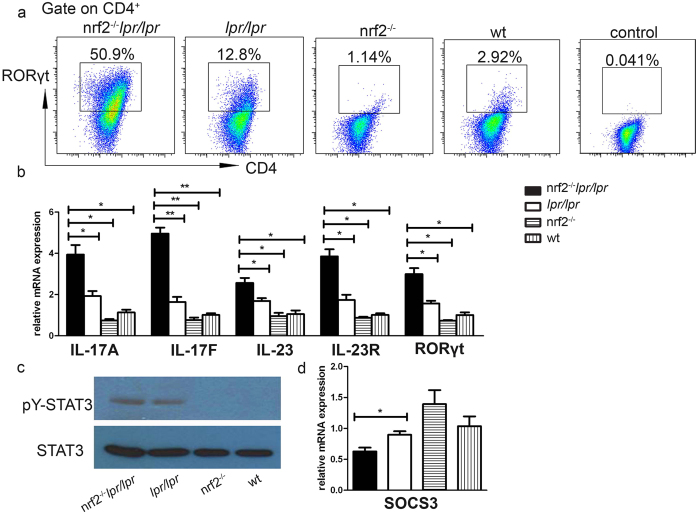
*In vitro* Th17 cell differentiation. Naïve CD4^+^ T cells were collected from mice of different genotypes and activated by plate-bound CD3 and CD28, then polarised into Th17 cells by treatment with IL-6 and TGF-β for 5 days. (**a**) Representative flow cytometry plots showing a higher proportion of RORγt^+^CD3^+^CD4^+^ Th17 cells in the *Nrf2*^−/−^*lpr*/*lpr* mice than in mice of other genotypes. (**b**) qRT-PCR analysis of Th17 cell-associated cytokines and (**d**) *Socs3* transcripts. (**c**) Western blot analysis of pY-STAT3 in naïve CD4^+^ T cells cultured in Th17 cell-conditioned media. Data values represent mean ± SEM. *p < 0.05, **p < 0.01, ***p < 0.001.

Through the analyses of cytokines in serum and the mRNA expression of cytokines in spleen and kidney, we suspected that Nrf2 may regulate the differentiation of the Th17 cells in the B6.*lpr*/*lpr* mice by stimulating IL-1β-mediated signalling. To test this further, we isolated the naïve CD4^+^ T cells and induced polarisation of the Th17 cells *in vitro*. We observed that the proportions of RORγt^+^CD4^+^ T cells and IL-17-producing cells markedly increased upon differentiation in Th17-conditioned culture media (Fig. 6a). Furthermore, the Th17-associated cytokines were more highly expressed in the B6.*Nrf2*^−/−^*lpr*/*lpr* cells than in cells with other genotypes (Fig. 6b). We also measured the phosphorylation of the transcription factor STAT3, which is required for the expression of RORγt and the development of Th17 cells, and found that the level of p-STAT3 increased in the B6.*Nrf2*^−/−^*lpr*/*lpr* mice (Fig. 6c). The mRNA expression of *Socs3*, which is the main negative regulator of p-STAT3, decreased in the B6.*Nrf2*^−/−^*lpr*/*lpr* mice (Fig. 6d). These results indicated that *Nrf2* deficiency induced the differentiation of Th17 cells by regulating the SOCS3/STAT3 signalling pathways.

## Discussion

In this study, we demonstrated that B6/*lpr* mice with a defect in *Nrf2* developed lupus-like autoimmune nephritis, which quickly advanced when compared to the B6/*lpr* mice (Figs 1 and 2). Interestingly, we also found changes in the proportion of Th17 cells among splenic CD4^+^ cells and the expression of Th17-related genes, such as *Il17, Il17f, Il23*, and *Il23r* (Fig. 5). Furthermore, a null mutation of *Nrf2* promoted the differentiation of Th17 cells *in vitro* (Fig. 6). These results indicated a relationship between the NRF2-mediated antioxidant pathway and Th17 cells during LN in B6.*Nrf2*^−/−^*lpr*/*lpr* mice.

Several studies have demonstrated that NRF2 is involved in the development of LN. A study in a human population identified that an *Nrf2* gene variation was associated with LN in childhood-onset SLE, and a GWAS analysis also determined the *Nrf2* locus as a region associated with susceptibility to SLE^22^,^23^. In NRF2-deficient mice, Yoh *et al*. found spontaneous LN development after 60 weeks of age^25^. Another study found that *Nrf2*-null mice developed an autoimmune disease with multiple organ pathologies that closely resembled human SLE^24^. In accordance with these studies, Jiang *et al*. demonstrated that pristane-induced *Nrf2*^−/−^ mice developed a lupus-like disease at 6 months of age that was regulated by the NF-κB and TGF-β1 signalling pathways^26^. However, conflicting data from a study by Morito *et al*. found that an *Nrf2* deficiency could extend lifespan and improve nephritis manifestations in *lpr* mice models, suggesting a protective effect of an *Nrf2* null mutation against SLE pathogenesis^28^. In *lpr* mice, the development of lupus-like autoimmune manifestations varies and is affected by the genetic background. In the MRL/Mp strain, *lpr* developed severe disease and the mice died by 6 months of age, while with the C57BL/6 or C3H background, *lpr* only developed mild nephritis by 14 months^27^,^31^. The clinical manifestations of ICR×MRL/*lpr* from the study of Morito *et al*. were similar to those in MRL/*lpr* mice, which had severe LN, and different from those in B6/*lpr* mice, which had slight renal damage at 6 months of age. The different genetic backgrounds used in those studies may be the reason for their conflicting results. These results revealed varied outcomes of null-mutant *Nrf2* dependent on genetic backgrounds, and caution should be exercised if using NRF2 as a therapeutic target in clinical practice.

It is well known that Th17 cells are involved in the regulation of LN pathogenesis through multiple mechanisms^19^. Th17 cells are a major type of pro-inflammatory T cells and many cytokines and transcription factors mediate the pathways that drive Th17 cell differentiation and promote Th17 immune responses^32^. Retinoic acid-related orphan receptor γ t (RORγt) is one of the key transcription factors in the development of Th17 cells^33^,^34^. Other transcription factors involved in Th17 differentiation include STAT3, which receives signals from IL-6 and IL-23 and regulates the expression of RORγt and IL-17 directly^35^,^36^. SOCS3, a member of the suppressor family of cytokine signalling, negatively regulates Th17 differentiation by down-regulating IL-23-mediated STAT3 phosphorylation^37^. Previous studies have demonstrated that enhancing NRF2 activation with triterpenoid compounds suppressed experimental autoimmune encephalitis by inhibiting Th17 function^38^. Another NRF2 inducer, sulforaphane, also inhibited antigen-specific Th17 responses and ameliorated the development of experimental autoimmune encephalitis^39^. However, no prior studies have demonstrated the effect of NRF2 on Th17 functions during the development of LN. In the present study with B6/*lpr* mice, we demonstrated that *Nrf2* deficiency elevated the serum concentration of IL-17 (Fig. 4), increased the percentage of Th17 cells among splenic lymphocytes, and increased the infiltration of Th17 cells into the kidneys (Figs 4 and 5). We also found that NRF2 inhibited Th17 differentiation by upregulating *Socs3* expression, which in turn decreased the phosphorylation of STAT3, a key modulator of Th17 differentiation (Fig. 6). These results demonstrated that NRF2 plays an important role in Th17 function during LN development by regulating the SOCS3/pSTAT3 axis. Moreover, we observed that the IL-1β concentration in serum was significantly higher in the *Nrf2*^−/−^*lpr/lpr* mice than in the other three strains (Fig. 4a), as was the mRNA expression of *Il1b* in the kidneys (Fig. 4b), macrophages (Fig. 4c), and splenocytes (Fig. 5). As in previous studies, IL-1β suppressed the expression of *Socs3* and enhanced STAT3 phosphorylation, which in turn enhanced Th17 differentiation^40^. Our data indicated that IL-1β could be another factor that augments Th17 differentiation and worsens kidney damage in the B6.*Nrf2*^−/−^*lpr/lpr* mice.

The NRF2-ARE signalling pathway, as the pivotal regulator of the antioxidant system, controls cellular oxidative stress by regulating the concentration of ROS with antioxidant enzymes. ROS, in turn, can activate many other pathways including the NF-κB pathway. Previous studies demonstrated that a deficit of *Nrf2* may increase oxidative stress and promote the development of LN^26^. In the pristane-induced mouse model of LN, mice with a defect of *Nrf2* had increased ROS production and strengthened NF-κB activation associated with renal damage, which could be prevented by NF-κB inhibitory peptides^26^. NF-κB is an important regulator of T cell differentiation, including all of the CD4^+^ T cell subsets^7^. During Th17 differentiation, NF-κB pathways directly enhanced *Rorγt* and *Il17* gene transcription^7^. In our study, we demonstrated that *Rorγt* and *Il17* expression dramatically increased in the *Nrf2*^−/−^*lpr*/*lpr* mice compared with that in other strains, and *Socs3* expression and STAT3 phosphorylation were also higher during Th17 differentiation (Fig. 6). However, we did not observe the same changes in the *Nrf2*-deficient mice (Figs 5 and 6), which indicated the possible relationship of enhanced NF-κB activation with the FAS signalling pathway for Th17 differentiation upon the null mutation of *Nrf2*. The interplay between ROS-enhanced NF-κB activation and FAS-mediated pathways in controlling the differential expression of Th17 subsets needs further study.

Cytokines also play a critical role in Th17 functions. IL-6, TGF-β, IL-1β, and IL-21 drive the differentiation of Th17 cells, and IL-23 promotes the expansion of Th17 cells^41^. In this study, we also found that the IL-1β level in serum significantly increased in the B6.*Nrf2*^−/−^*lpr*/*lpr* mice, which may be another factor to promote Th17 function and LN development (Fig. 4). IL-1β also dramatically increased in splenic lymphocytes and slightly increased in activated macrophages from the peritoneal lavage, which indicated multiple sources of IL-1β production (Figs 4 and 5). IL-1β production can be controlled through two different mechanisms. One mechanism is driven at the transcriptional level by NF-κB, with the subsequent stimulation of *Il1b* gene expression. Another is induced at the post-transcriptional level and is associated with activation of the inflammasome, which converts the immature cytokine pro-IL-1β to the active form^42^. As ROS-mediated oxidative stress regulated the NF-κB pathway and inflammasome activation, *Nrf2* deficiency increased IL-1β production through those two mechanisms by increasing ROS levels. Further studies are needed to determine which mechanism, the NF-κB pathway or the regulation of inflammasome activation, is the most responsible for IL-1β production.

In summary, this study demonstrated that Th17 cells were involved in the regulation of *Nrf2* deficiency that promoted the development of LN. We also found a new mechanism of the NRF2-mediated regulation of LN development. In addition, the SOCS3/STAT3 pathways and IL-1β were both involved in mediating the effect of NRF2 on controlling Th17 differentiation. These findings suggested that NRF2 has a role in LN development and different therapeutic strategies could be developed for patients with different genetic backgrounds. Therefore, the regulation of Th17 differentiation by NRF2 could be considered as a new therapeutic target for the treatment of subclinical LN patients.

## Materials and Methods

### Ethics statement

This study was carried out in strict accordance with the guidelines of the Institute for Laboratory Animal Research of Sun Yat-sen University Laboratory Animal Center (Guangzhou, China). The protocol was approved by the Laboratory Animal Welfare and Ethics Committee of the Sun Yat-sen University [Permit Number: SYXK (YUE) 2011-0112].

### Mice

*Nrf2*-deficient mice were purchased from the Jackson Laboratory (Bar Harbor, ME, USA) from the 129 × B6 F1 background, and then backcrossed into a C57BL/6 strain for 10 generations and designed as B6.*Nrf2*^−/−^ mice. The B6/*lpr* mice were purchased from the National Resource Center for Mutant Mice of China (Nanjing, China), and crossed with B6.*Nrf2*^−/−^ mice to obtain double-mutant mice designed as the B6.*Nrf2*^−/−^*lpr/lpr* mice. All mice were raised in a specific pathogen-free facility under a 12-h light–12-h dark cycle at 22 °C in Sun Yat-sen University Laboratory Animal Center (Guangzhou, China). Knockout (KO) mice were produced by heterozygous-heterozygous mating. We used WT littermates for comparison. The genotype was confirmed by PCR using primers as shown in Table S1.

### Enzyme-linked immunosorbent assay (ELISA) for serum anti-dsDNA antibody measurement

Serum anti-dsDNA antibody levels were measured by ELISA. The optical density at 450 nm was read using a microplate reader (Multiskan MK3, Thermo Fisher Scientific, Waltham, MA, USA). Serum was diluted 1:100 in phosphate-buffered saline (PBS), and then diluted two-fold seven times. ELISA plates were pre-treated with polylysine (2 μg/mL) at 37 °C for 2 h. The plate was then washed with PBS containing 0.1% Tween 20 three times (PBST). After washing, ELISA plates were coated with mouse dsDNA solution (5 μg/mL) at 4 °C overnight. Afterward, the plates were washed and blocked with 5% fetal bovine serum at 37 °C for 2 h. After washing, serial dilutions of test sera were added to each well and incubated at 37 °C for 1.5 h. The plates were washed three times with PBST and incubated with horseradish peroxidase-conjugated goat-anti-mouse IgG (Sigma–Aldrich, MO, USA) at 37 °C for 45 min. After washing the plates three times, 3,3′,5,5′-tetramethylbenzidine single solution (Thermo Fisher Scientific) was added to each well and the colorimetric reaction was allowed to develop at room temperature for 3 min. The reaction was stopped with 2 M H_2_SO_4_ and then the optical density was determined.

### Measurement of blood urea nitrogen and total protein

Each mouse was bled while under ether anaesthesia, and sera were separated by centrifugation and stored at −80 °C until use. The levels of blood urea nitrogen and total protein in mice serum were measured by an automated analyser for routine laboratory testing (7180 Automatic Biochemical Analyser, Hitachi, Tokyo, Japan).

### Histopathologic analysis of renal tissues

Kidneys were soaked in 4% formalin overnight at room temperature, embedded in paraffin, and sectioned at 4-μm thickness. Sections were stained with hematoxylin and eosin (H&E) or periodic acid–Schiff (PAS) for histopathologic examination under a light microscope. Sections were visualised by light microscopy at 400× magnification. Six sections were histologically scored as previously described^43^.

### Immunofluorescence detection

For the examination of glomerular immune complex deposits, kidney sections were stained as previously described^44^ with the following antibodies: Alexa Fluor 488 goat anti-mouse IgM (μ chain) and Alexa Fluor 488 goat anti-mouse IgG (H+L) (Invitrogen, Carlsbad, CA, USA).

### Flow cytometry

Lymph nodes and spleen were disintegrated into single cells as previously described^45^ and stained for surface markers with the following antibodies: TCR-β (H57-597), B220 (R43-6B2), CD4 (RM4-5), and CD8 (53-6.7), and all were purchased from BD Biosciences (Franklin Lakes, NJ, USA) or eBioscience (San Diego, CA, USA).

Spleen cells were stimulated as previously described^46^, and then stained for intracellular cytokines using a Mouse FOXP3 Buffer Set (BD Biosciences). The following fluorochrome-conjugated antibodies were used: CD4 (GK1.5), CD25 (7D4), CD3 (145-2C11), FOXP3 (FJK-16s), RORγt (AFKJ-9), IFNγ (XMG1.2), and IL-17 (eBio17B7), and all were purchased from BD Biosciences or eBioscience.

Data was collected with a FACSCanto instrument (BD Biosciences).

### T cell differentiation *in vitro*

CD4^+^CD25^−^ T cells were isolated from spleens of 6-month-old mice as previously described^47^. Cells were purified using a MACS magnetic column with a CD4^+^ T cell enrichment kit (eBioscience). Then, cells were stimulated with a plate-bound anti-CD3e antibody (5 μg/mL, eBioscience) and anti-CD28 antibody (2 μg/mL, eBioscience). For Th17 cell polarisation, we added exogenous cytokines and the following antibodies: IL-6 (30 ng/mL, R&D Systems, Minneapolis, MN, USA); TGF-β (5 ng/mL, R&D Systems); IL-1β (20 ng/mL, R&D Systems); anti-IL-4 antibody (10 μg/mL; eBioscience); and anti-IFN-γ antibody (10 μg/mL, eBioscience). Cells were cultured in a culture incubator at 37 °C in a humidified atmosphere of 5% CO_2_, and after 5 days, we measured the proportion of Th17 cells among all cells by flow cytometry and qRT-PCR.

### Immunoblot analysis

We used western blot analysis to evaluate the phosphorylation of STAT3 in Th17-polarising cells. Cells were lysed in 200 μl of lysis buffer (150 mM NaCl, 50 mM Tris, 4 mM KCl, 1 mM MgCl_2_, 1 mM Na_3_VO_4_, 10% glycerol, 1% Nonidet P-40, and protease inhibitors). Primary antibodies were as follows: anti-STAT3 (4904; Cell Signaling Technology, Danvers, MA, USA), anti-phospho-STAT3 at Tyr705 (9145; Cell Signaling Technology). Horseradish peroxidase-conjugated anti-rabbit (7074; Cell Signaling Technology) was used for the tested target proteins with ECL Chemiluminescent Substrate Reagent Kit (WP20005, Thermo Fisher Scientific).

### RNA isolation and quantitative RT-PCR

Total RNA samples from macrophages, spleen, and kidney were extracted using TRIzol reagent (Invitrogen). Total RNA was reverse transcribed into cDNA using the PrimeScript RT Reagent Kit (Takara Bio, Kusatsu, Japan). This was followed by real-time quantification using GoTaq qPCR Master Mix (Promega, Madison, WI, USA). The PCR control we used was the housekeeping gene glyceraldehyde-3-phosphate dehydrogenase (*Gapdh*). The average of the values obtained for WT mice was set as the baseline. Quantitative RT-PCR was performed using the primers listed in Table S2.

### Cell culture

Spleen lymphocytes and macrophages were cultured in RPMI 1640 (Invitrogen) with 10% fetal bovine serum (HyClone, GE Healthcare, Little Chalfont, UK) supplemented with 1 mM sodium pyruvate, 2 mM glutamine, 50 μM 2-mercaptoethanol, and 100 U/mL penicillin/streptomycin. Cells were cultured in a culture incubator at 37 °C in a humidified atmosphere of 5% CO_2_.

### Macrophage isolation and culture

Macrophages were isolated by peritoneal lavage as previously described^48^ and cultured for 24 h with immune stimulation by lipopolysaccharides (10 μg/mL, Sigma–Aldrich) as previously described^48^,^49^.

### Mouse serum cytokine assay

To measure the cytokine levels in serum, we used a Bio-Plex Pro Mouse Cytokine 23-Plex panel (Bio-Rad Laboratories, Hercules, CA, USA). Array analysis was performed using the Bio-Plex Protein Array system (Bio-Rad Laboratories) following the manufacturer’s instructions.

### Statistical analysis

Statistical significance was determined by unpaired Student’s *t*-tests using GraphPad Prism 5 (GraphPad Software Inc., San Diego, CA, USA). The Kaplan–Meier method was used to analyse the survival rate. Significance was ascribed when *p* < 0.05.

## Additional Information

**How to cite this article**: Zhao, M. *et al*. Nuclear Factor Erythroid 2-related Factor 2 Deficiency Exacerbates Lupus Nephritis in B6/*lpr* mice by Regulating Th17 Cell Function. *Sci. Rep.* **6**, 38619; doi: 10.1038/srep38619 (2016).

**Publisher's note:** Springer Nature remains neutral with regard to jurisdictional claims in published maps and institutional affiliations.

## Supplementary Material

Supplementary Dataset

## Figures and Tables

**Table 1:** FACS analysis of spleen cells from 6-month-old mice (mean ± SEM).

Genotype	*Nrf2*^−/−^ *lpr/lpr n* = 9	*lpr/lpr n* = 9	*Nrf2*^−/−^ *n* = 9	WT *n* = 9
Total cell number (×10^6^)	161.6 ± 26.35	98.67 ± 18.18	59.11 ± 14.48	56.78 ± 8.00
T cell number (×10^6^)	41.13 ± 7.55	28.89 ± 5.71	21.17 ± 4.93	17.29 ± 2.98
B cell number (×10^6^)	48.52 ± 9.19	37.77 ± 7.69	21.91 ± 5.92	20.70 ± 3.78
CD4^+^ T cell number (×10^6^)	33.62 ± 4.00	20.65 ± 4.14	12.96 ± 2.97	10.45 ± 1.71
CD8^+^ T cell number (×10^6^)	6.69 ± 1.80	5.73 ± 1.19	6.70 ± 1.59	11.46 ± 3.32
