# Supplementary material for: Nuclear Factor Erythroid 2-related Factor 2 Deficiency Exacerbates Lupus Nephritis in B6/lpr mice by Regulating Th17 Cell Function
Source: Sci Rep. 2016 Dec 12;6:38619. doi: 10.1038/srep38619 (PMC5150244; doi:10.1038/srep38619)
Supplement: Supplementary Dataset [file srep38619-s1.doc]

**Nuclear Factor Erythroid 2-related Factor 2 Deficiency Exacerbates Lupus Nephritis in B6/*lpr* mice by Regulating Th17 Cell Function**

Mei Zhao^1,2^, Huanpeng Chen^1,2^, Qingfeng Ding^1,2^, Xiaoxie Xu^1,2^, Bolan Yu^3^, Zhaofeng Huang^1,2,4,*^

^1^Institute of Human Virology, Zhongshan School of Medicine, Sun Yat-sen University, Guangzhou, China

^2^Key Laboratory of Tropical Diseases Control, Sun Yat-sen University, Ministry of Education in China, Guangzhou, China

^3^Key Laboratory for Major Obstetric Diseases of Guangdong Province, Third Affiliated Hospital of Guangzhou Medical University, Guangzhou, China.

^4^Department of Biochemistry, Zhongshan School of Medicine, Sun Yat-sen University.

*****Corresponding author: Dr. Zhaofeng Huang, address is: N1311 Rm, No.10 Bld, 74 Zhongshan 2^nd^ Rd, Guangzhou, 510080, China, Tel: 8620-87335529, Fax: 8620-87332588, E-mail: [hzhaof@mail.sysu.edu.cn](mailto:hzhaof@mail.sysu.edu.cn)

**Table S1**

| **Primer type** | **Sequence** |
| --- | --- |
| nrf2 common  nrf2 mutant  nrf2 wild type  fas common  fas mutant  fas wild type | 5’- GCCTGAGAGCTGTAGGCCC-3’  5’- GACAGTATCGGCCTCAGGAA-3’  5’- GGAATGGAAAATAGCTCCTGCC-3’  5’- GTAAATAATTGTGCTTCGTCAG-3’  5’- TAGAAAGGTGCACGGGTGTG-3’  5’- CAAATCTAGGCATTAACAGTG-3’ |

**Sequences of primers for PCR to determine mouse genotypes**

**Table S2**

| **Target gene** | **Forward sequence** | **Reverse sequence** |
| --- | --- | --- |
| IL-6 | 5’-CCAAGAGGTGAGTGCTTCCC-3’ | 5’-CTGTTGTTCAGACTCTCTCCCT-3’ |
| IL-23 | 5’-ATGCTGGATTGCAGAGCAGTA-3’ | 5’-ACGGGGCACATTATTTTTAGTCT-3’ |
| IL-1β | 5’-GAAATGCCACCTTTTGACAGTG-3’ | 5’-TGGATGCTCTCATCAGGACAG-3’ |
| TNF-α | 5’-CCCTCACACTCAGATCATCTTCT-3’ | 5’-GCTACGACGTGGGCTACAG-3’ |
| G-CSF | 5’-ATGGCTCAACTTTCTGCCCAG-3’ | 5’-CTGACAGTGACCAGGGGAAC-3’ |
| GM-CSF | 5’-GGCCTTGGAAGCATGTAGAGG-3’ | 5’-GGAGAACTCGTTAGAGACGACTT-3’ |
| IL-23R | 5’-TTCAGATGGGCATGAATGTTTCT-3’ | 5’-CCAAATCCGAGCTGTTGTTCTAT-3’ |
| ROR_γt_ | 5’-AAACTTGACAGCATCTCGGGA-3’ | 5’-TGCAGGAGTAGGCCACATTACA-3’ |
| IL-17 | 5’- CTCCAGAAGGCCCTCAGACTAC -3’ | 5’- AGCTTTCCCTCCGCATTGACACAG -3’ |
| IL-17F | 5’-GAGGATAACACTGTGAGAGTTGAC-3’ | 5’-GAGTTCATGGTGCTGTCTTCC-3’ |
| TGF-β1 | 5’-ACCATGCCAACTTCTGTCTG-3’ | 5’-CGGGTTGTGTTGGTTGTAGA-3’ |
| SOCS3 | 5’- GGCCCTTCTCCAGGACAGA-3’ | 5’- GCTGATCATGGCTGGGTTGT-3’ |
| Gapdh | 5’-TGGTGAAGGTCGGTGTGAAC-3’ | 5’-CCATGTAGTTGAGGTCAATGAAGG-3’ |

**Sequences of primers for quantitative RT-PCR**
